# Prevalence of methicillin-resistant *Staphylococcus aureus* (MRSA) in retail food in Singapore

**DOI:** 10.1186/s13756-017-0255-3

**Published:** 2017-09-08

**Authors:** Kyaw Thu Aung, Li Yang Hsu, Tse Hsien Koh, Hapuarachchige Chanditha Hapuarachchi, Man Ling Chau, Ramona Alikiiteaga Gutiérrez, Lee Ching Ng

**Affiliations:** 1 Environmental Health Institute, National Environment Agency, 11 Biopolis Way, #04-03/04, Helios Block, Singapore, 138667 Singapore; 2 Department of Infectious Diseases, Tan Tock Seng Hospital, 11 Jalan Tan Tock Seng, Singapore, 308433 Singapore; 3 Diagnostic Bacteriology, Pathology Department, Singapore General Hospital, 20 College Road, Academia, Level 7, Diagnostic Tower, Singapore, 169856 Singapore; 4 School of Biological Sciences, College of Science, Nanyang Technological University, 60 Nanyang Drive, Singapore, 657551 Singapore; 5 School of Chemical and Biomedical Engineering, College of Engineering, Nanyang Technological University, 62 Nanyang Drive, Singapore, 637459 Singapore

**Keywords:** Methicillin-resistant *Staphylococcus aureus* (MRSA), Retail food, Food contact surface, Antibiotic resistance, Enterotoxin genes, Panton-valentine leukocidin (*pvl*) gene

## Abstract

We characterised 227 *Staphylococcus aureus* isolates from retail food and food handlers’ gloves samples obtained through food surveillance and risk assessment studies between 2011 and 2014. Of 227 isolates, five (2.2%) were methicillin-resistant and belonged to sequence types ST80 (*n* = 3) and ST6 (*n* = 2). All five isolates belonged to SCC*mec* type IV, were Panton-Valentine leukocidin *(pvl)*-negative and staphylococcal enterotoxin genes-positive. Resistance to azithromycin was found in ST80 isolates, in addition to resistance to beta-lactams. Our finding of two clinically relevant methicillin-resistant *S. aureus* (MRSA) strains (ST80 and ST6) in ready-to-eat food and food contact surfaces at retail in Singapore suggests food and food contact surfaces as potential environmental sources of MRSA in the community.

Dear Editor,

Methicillin-resistant *Staphylococcus aureus* (MRSA) has been recognised as an important nosocomial pathogen and has reportedly been associated with foodborne illnesses [1–3]. MRSA has also recently been listed as one of the high-priority antibiotic-resistant pathogens as ranked by the World Health Organisation. In Singapore, surveillance and control programmes of MRSA have been established in various hospitals [4, 5]. However, there is a substantial lack of information on the prevalence of MRSA in food, including at retail. Such information would be useful to better understand the risk of exposure to MRSA through food, particularly ready-to-eat food, in contrast with the more typical known transmission route via contact. Our findings provide preliminary insights on the extent of the spread of MRSA in ready-to-eat food in Singapore.

In this study, we characterised 227 coagulase-positive *Staphylococcus aureus* strains isolated from retail food and swabs taken from food handlers’ gloves. These isolates and samples were obtained from food surveillance and risk assessment studies conducted between 2011 and 2014. The isolates were confirmed to be *S. aureus* using coagulase rabbit plasma (Remel), and conventional PCR for the detection of *femA* gene with modified primers (F- GATCATTTATGGAAGATACGTCAG, R- GATAAAGAAGAAACCAGCAGAGATAG) and PCR conditions from previous studies [6, 7]. To understand the occurrence of methicillin resistance among these *S. aureus* isolates, we used *mecA*-PCR for the detection of MRSA, followed by PBP2 latex agglutination test (Oxoid) and disc diffusion with Cefoxitin 30 μg for confirmation. MRSA strains were further characterised by Multi-Locus Sequence Typing (MLST), staphylococcal Protein A (*spa*) typing, and staphylococcal cassette chromosome (SCC*mec*) typing. Strains were also analysed for the presence of virulence genes: staphylococcal enterotoxin (*sea, seb, sec, sed, see*, *seg, seh, sei, sej, sek, sel, sem*, *seo* and *sep*), Panton-Valentine leukocidin (*pvl*), exfoliative toxin (*eta*, *etb* and *etd*), and toxic shock syndrome toxin (*tsst*-1) genes [7, 8]. Antibiotic susceptibility testing was performed and interpreted according to the Clinical and Laboratory Standards Institute (CLSI) guideline [9].

Of the 227 *S. aureus* isolates*,* five (2.2%) from two food stalls were methicillin-resistant (Table 1) and belonged to Sequence Types (ST) 80 (spa type t1198) (isolated from sliced onion, prawn fritters, fried egg) and ST6 (spa type t304) (from swabs of food handlers’ gloves) [7]. All five isolates belonged to SCC*mec* type IV, and were *pvl*-negative. Unlike SCC*mec* types I-III strains which are often associated with nosocomial infections, strains belonging to SCC*mec* type IV are capable of colonising healthy individuals and may be community-associated. Some SCC*mec* type IV strains may be associated with livestock, suggesting a possible transmission between human and food-producing animals [10–12]. In Singapore, community-associated (CA) MRSA strains previously reported were isolated from clinical samples and belonged mainly to ST30, ST59 and ST772 [13]. To our knowledge, no ST6 strain has been reported in Singapore, whereas a ST80 CA-MRSA strain was previously reported in a local hospital in 2003, from a patient with a chin abscess [8]. *S. aureus* ST80, belonging to Clonal Complex 80 (CC80), was first reported in Denmark in 1993 and has been recognised as a major clone of CA-MRSA, widely spread across Europe [14–16]. Most ST80 strains reported elsewhere belong to SCC*mec* type IV, are *pvl*-positive, and are usually associated with severe skin/soft tissue infections and necrotising pneumonia [17]. Nonetheless, *pvl*-negative ST80 strains have also been reported in clinical cases overseas [18, 19]. ST6 MRSA is a double-locus variant of CC5, which is one of the five CCs from which major epidemic MRSA isolates are believed to have emerged [20]. ST6 MRSA was previously reported in human cases and carriers in Australia, Oman and United Arab Emirates (UAE) [16, 21, 22]. In particular, ST6 SCC*mec* type IV (t304) *pvl-*negative MRSA, a strain susceptible to non-beta-lactams, was previously isolated from patients overseas, suggesting that the ST6 strain isolated in our study may be capable of causing human infection [23]. In addition, CC6-ST6-t304 strains have previously been isolated from human (MSSA and MRSA), feral cat (MRSA) and camel (MSSA) suggesting that the strain may be transmissible across different host species [19, 24–26].

**Table 1:** Phenotypic and genotypic characteristics of methicillin resistant *Staphylococcus aureus* (MRSA) isolated from retail food and contact surfaces in Singapore. *MLST* multi locus sequence typing, *spa* staphylococcal protein A, *pvl* panton-valentine leukocidin, *se* staphylococcal enterotoxin, *et* exfoliative toxin, *tsst-1* toxic shock syndrome toxin-1, *AMC* amoxycillin-clavulanic acid, *AMP* ampicillin, *FOX* cefoxitin, *CRO* Ceftriaxone, *P* penicillin, *AK* amikacin, *AZM* azithromycin, *CIP* ciprofloxacin, *C* chloramphenicol, *CN* gentamicin, *NOR* norfloxacin, *TE* tetracycline, *SXT* trimethoprim-sulphamethoxazole

Food stall	Year of isolation	Source	Molecular typing			pvl gene	Staphylocooccal enterotoxin genes														Exfoliative toxin genes			tsst -1	Beta lactams					Non beta lactams							
			MLST	spa	SCCmec		sea	seb	sec	sed	see	seg	seh	sei	sej	sek	sel	sem	seo	sep	eta	etb	etd		AMC	AMP	FOX	CRO	P	AK	AZM	CIP	C	CN	NOR	TE	SXT
A	2011	Sliced Onion	ST80	t1198	IV	-	-	+	-	-	-	-	-	-	-	+	-	-	-	-	-	-	+	-	R	R	R	R	R	S	R	S	S	S	S	S	S
A	2011	Prawn fritters	ST80	t1198	IV	-	-	+	-	-	-	-	-	-	-	+	-	-	-	-	-	-	+	-	R	R	R	R	R	S	R	S	S	S	S	S	S
A	2011	Fried egg	ST80	t1198	IV	-	-	+	-	-	-	-	-	-	-	+	-	-	-	-	-	-	+	-	R	R	R	R	R	S	R	S	S	S	S	S	S
B	2013	Gloves (inner surface)	ST6	t304	IV	-	+	-	-	-	-	-	-	-	-	-	-	-	-	-	-	-	-	-	R	R	R	R	R	S	S	S	S	S	S	S	S
B	2013	Glove (outer surface)	ST6	t304	IV	-	+	-	-	-	-	-	-	-	-	-	-	-	-	-	-	-	-	-	R	R	R	R	R	S	S	S	S	S	S	S	S

In this study, we detected enterotoxin genes *seb*, *sek*, and exfoliative toxin gene *etd* in ST80 strains; and enterotoxin gene *sea* in ST6 strains [7]. No other toxin genes were detected. Enterotoxins *sea* and *seb* are known to cause approximately 90% of staphylococcal food poisoning worldwide [27]. Enterotoxin *sek* is considered a staphylococcal enterotoxin-like protein, though its ability to cause food poisoning has yet to be demonstrated [28]. *etd* is an exfoliative toxin serotype known to be associated with mild forms of cutaneous infections such as abscesses and furuncles [29]. The presence of enterotoxin genes (*sea* and *seb*) in these MRSA isolates suggests the isolates’ potential to produce toxins, and cause staphylococcal food poisoning, if allowed to grow in sufficient numbers in food. Antimicrobial susceptibility results showed that all 5 MRSA isolates were phenotypically susceptible to amikacin, ciprofloxacin, chloramphenicol, gentamicin, norfloxacin, tetracycline, and trimethoprim/sulfamethoxazole. Susceptibility to at least 3 non-beta-lactams in MRSA is used as a proxy to define community-associated strains [30]. We found ST80 isolates resistant to azithromycin in addition to the beta-lactams tested (Table 1). An emerging trend of macrolide resistance has been reported in CA-MRSA from the human and livestock sectors [11, 31]. MRSA with resistance to additional antibiotic classes is a concern, and may reflect the increasing use of these antibiotics in the local clinical practice, which warrants further investigation.

In conclusion, we report two clinically relevant MRSA strains (ST80 and ST6) in ready-to-eat food and food contact surfaces at retail. Humans (food handlers), rather than animals, were likely the sources of contamination. Our limited findings suggest ready-to-eat food and food contact surfaces as potential environmental sources for colonisation and spread of MRSA in the community. To date, little is known about the transmission of MRSA infections through food and food contact surfaces, however their possible roles in the dissemination of specific MRSA lineages cannot be ruled out. The data warrant a more comprehensive and integrated (farm-to-hospital approach) surveillance of MRSA in Singapore and elsewhere.

## Abbreviations

AK: Amikacin
AMC: Amoxycillin-clavulanic acid
AMP: Ampicillin
AZM: Azithromycin
C: Chloramphenicol
CA-MRSA: Community-associated methicillin-resistant *Staphylococcus aureus*
CIP: Ciprofloxacin
CN: Gentamicin
CRO: Ceftriaxone
*Et*: Exfoliative toxin
FOX: Cefoxitin
*mecA*: Gene encoding methicillin-resistant-*S. aureus*-specific-penicillin-binding protein
MLST: Multi-Locus Sequence Typing
MRSA: Methicillin-resistant *Staphylococcus aureus*
NOR: Norfloxacin
P: Penicillin
PBP2: Penicillin binding protein 2
*Pvl*: Panton-valentine leukocidin
SCC*mec*: Staphylococcal cassette chromosome
*Se*: Staphylococcal enterotoxin
Spa: Staphylococcal Protein A
ST: Sequence type
SXT: Trimethoprim-Sulphamethoxazole
TE: Tetracycline
*Tsst*: Toxic shock syndrome toxin
